# BLT2 is expressed in PanINs, IPMNs, pancreatic cancer and stimulates tumour cell proliferation

**DOI:** 10.1038/sj.bjc.6604655

**Published:** 2008-09-09

**Authors:** R Hennig, T Osman, I Esposito, N Giese, S M Rao, X-Z Ding, W-G Tong, M W Büchler, T Yokomizo, H Friess, T E Adrian

**Affiliations:** 1 Department of Surgery, Technische Universität München, Munich, Germany; 2 Department of Surgery, University of Heidelberg, Heidelberg, Germany; 3 Institute of Pathology, Technische Universität München, Munich, Germany; 4 Institute of Pathology, Helmholtz Zentrum München, Neuherberg, Germany; 5 Department of Pathology, Feinberg School of Medicine, Northwestern University, Chicago, IL, USA; 6 Department of Surgery, Feinberg School of Medicine, Northwestern University, Chicago, IL, USA; 7 Department of Medical Biochemistry, Graduate School of Medical Sciences, Kyushu University, Fukuoka, and CREST of JST, Japan; 8 Department of Physiology, United Arab Emirates University, Faculty of Medicine and Health Sciences, Al Ain, UAE

**Keywords:** BLT2, PanIN, IPMN, pancreatic cancer, leukotriene B_4_

## Abstract

Pancreatic cancer has an abysmal prognosis. Targets for early detection, prevention and therapy are desperately needed. Inflammatory pathways have an important impact on tumour growth and progression. Expression of BLT2 (the second leukotriene B_4_ receptor) was investigated by real-time RT–PCR and immunohistochemistry. Cell proliferation was studied after stable transfection with BLT2, after treatment with siRNA and Compound A as an agonist. BLT2 is expressed in all pancreatic cancer cell lines. Results from real-time RT–PCR revealed significant overexpression of BLT2 in malignant intraductal papillary mucinous neoplasias (IPMNs) and pancreatic adenocarcinoma. Intense staining was evident in IPMNs, infiltrating tumour cells and advanced pancreatic intraepithelial neoplasias (PanINs) but not in normal ductal cells. Overexpression of BLT2 as well as stimulation of Colo357, Panc-1 and AsPC1 cells with Compound A caused a significant increase in tumour cell proliferation, an effect reversed after siRNA treatment. This study demonstrates for the first time the expression of BLT2 in the pancreas and overexpression in pancreatic cancers and malignant IPMNs in particular. Upregulation of BLT2 is already evident in precursor lesions (PanINs, IPMNs). Overexpression of this receptor leads to significant growth stimulation. Therefore, we suggest BLT2 as a new target for chemoprevention and therapy for pancreatic cancer.

Pancreatic cancer is the fourth leading cause of cancer death (after lung, colon and breast) in the United States and the incidence of this disease has not declined (Jemal *et al*, 2006). Indeed it has increased in African Americans as well as in the Japanese over recent years (Oomi and Amano, 1998; Lin *et al*, 1998; Stat bite, 2002; Qiu *et al*, 2005; Luo *et al*, 2007). The mortality of pancreatic cancer almost equals its incidence and most patients die within 6 months because of late diagnosis and lack of effective therapies (Howard, 1996; Jemal *et al*, 2006). Potentially curative surgery is an option only in 9–20% of patients, because of existing liver metastases or the invasion of major blood vessels (Chua and Cunningham, 2005). However, even in this selected group 2- and 5-year survival rates are at about 40 and 20%, respectively and the median survival time is 20 months when patients receive adjuvant chemotherapy (Neoptolemos *et al*, 2004; Stocken *et al*, 2005). Many patients have recurrent disease 12 months after surgery because of tumour recurrence or metastatic tumour progression (Ahrendt and Pitt, 2002; Chua and Cunningham, 2005). Therefore, identification of risk factors and early diagnostic markers as well as new therapeutic approaches are desperately needed so that the disease can be prevented or detected at an early, non-invasive stage. It is believed that ductal pancreatic adenocarcinomas originate from ductal cells. Pancreatic intraepithelial neoplasias (PanINs) are histologically defined lesions in the small ducts and ductules that are thought to be the precursors of pancreatic cancer (Hruban *et al*, 2001). PanINs are potentially an ideal target for chemoprevention and so a specific marker for these lesions is likely to be useful for early diagnosis (Hruban *et al*, 2000, 2001).

Epidemiological and animal studies suggest that a high intake of polyunsaturated fatty acids (PUFAs) is associated with an increased incidence and growth of tumours at several specific organ sites including pancreas, colon, breast and prostate (Rose, 1997; Woutersen *et al*, 1999a, 1999b). A recent review pointed out the important role of cyclooxygenase and lipoxygenase pathways in fat metabolism and in the regulation of pancreatic cancer cell proliferation and survival (Ding *et al*, 2003). Cyclooxygenase-2 (COX-2) plays an important role in carcinogenesis, is upregulated in 56–90% of pancreatic adenocarcinomas and 65% of PanINs and frequent use of aspirin seems to decrease the risk of pancreatic cancer (Hitt, 2002; Maitra *et al*, 2002; Ding *et al*, 2003). The 5-lipoxygenase (5-LOX) pathway seems to have an even more important function in pancreatic carcinogenesis (Ding *et al*, 2003). Leukotriene B_4_ (LTB_4_) is a downstream metabolite of 5-LOX and stimulates pancreatic cancer cell growth through ERK1/2 phosphorylation, which can be inhibited by an LTB_4_ receptor antagonist (Tong *et al*, 2002). However, LY293111 is not specific as it inhibits 5-LOX activity and is also a PPAR*γ* agonist. Recently, we have reported that the LTB_4_ receptor 1 (BLT1) is overexpressed in pancreatic cancer cells and tissues as well as islets adjacent to the tumour (Hennig *et al*, 2002). LTB_4_ receptors are G-protein-coupled receptors which belong to the chemoattractant receptor group (Yokomizo *et al*, 2001). BLT1 was isolated and cloned by Yokomizo's group in 1997 (Yokomizo *et al*, 1997). Three years later they identified a second, low-affinity receptor for LTB_4_ (BLT2), with 45% homology with BLT1 at the amino acid level (Yokomizo *et al*, 2000). The open reading frame (ORF) of BLT2 is localised upstream of BLT1 and contains the promoter region of BLT1. This represents a very rare case of a ‘promoter in ORF’ in higher eukaryocytes, the physiological significance of which has not been determined (Yokomizo *et al*, 2000). BLT1 (352 amino acids) is almost exclusively expressed in peripheral leukocytes with low level expression in human spleen and thymus (Yokomizo *et al*, 1997). However, BLT2 (358 amino acids) is expressed in spleen, liver, ovary and leukocytes and low levels of its mRNA have been demonstrated in many tissues (Yokomizo *et al*, 2000). Recently, expression of BLT2 in dendritic cells was described, speculating on a regulatory role in dendritic cell trafficking during induction of immune responses (Shin *et al*, 2006). Moreover, BLT2 was found in mast cells, possibly mediating recruitment and accumulation of mast cells in areas of inflammation (Lundeen *et al*, 2006). Further studies showed by *in situ* hybridisation that expression of BLT2 is significantly upregulated in a variety of human cancers (Yoo *et al*, 2004). In addition, it has been suggested that BLT2 is responsible for LTB_4_-induced generation of reactive oxygen species (ROS) through Rac/ERK and that this receptor is involved in cytokine-induced differentiation and expansion of haematopoietic stem cells (Woo *et al*, 2002; Chung *et al*, 2005). Stem cell factor and TNF-*α* are suggested to regulate BLT2 expression (Lundeen *et al*, 2006; Qiu *et al*, 2006).

There is a strong relationship between chronic inflammation and development of cancers of the gastrointestinal tract, including oesophagitis with Barrett's metaplasia and ultimately oesophageal adenocarcinoma, inflammatory bowel disease with colon cancer, and chronic pancreatitis with pancreatic cancer (Orlando, 2002; Itzkowitz and Yio, 2004; Whitcomb, 2004). The lipoxygenase pathway in particular has been linked with development and growth of pancreatic adenocarcinoma, whereas inhibitors of this pathway, including LY293111, inhibit growth of the cancer (Hennig *et al*, 2002; Tong *et al*, 2002; Ding *et al*, 2003). As the function of the second LTB_4_ receptor in pancreatic cancer is not known, we investigated the expression and biological importance of BLT2 in human pancreatic cancer cells and tissues. This study follows our previous work investigating the function of the 5-lipoxygenase pathway in pancreatic carcinogenesis.

## Materials and methods

### Materials

RPMI-1640, penicillin–streptomycin solution, trypsin-EDTA solution were purchased from Gibco (Invitrogen, Karlsruhe, Germany). Fetal bovine serum (FBS) was from PAA (PAA Laboratories, Cölbe, Germany).

Compound A and its methyl ester derivative (Compound B=control) are gifts from Ono Pharmaceutical Co. Ltd. (Ono Pharmaceutical Co., Ltd., Chuoku, Osaka, Japan). The BLT2 antibody and LTB_4_ were purchased from Cayman Chemical Co. (Ann Arbor, MI, USA).

BLT2 siRNA was purchased from Ambion Inc. (Ambion, Austin, TX, USA) and negative control siRNA from Qiagen (Qiagen, Hilden, Germany).

### Immunohistochemistry for BLT2

Tissue samples from 10 patients with chronic pancreatitis and 10 with pancreatic ductal adenocarcinoma were examined. As shown in Table 1, 12 of these surgical pancreatic specimens showed PanIN lesions. Six specimens showed PanIN-1a and 1b lesions, five had PanIN-2 and six had PanIN-3 lesions. Ten pancreas specimens from multi-organ donors were included as controls. However, one of them contained PanIN-1a lesions.

In addition, seven specimens with benign intraductal papillary mucinous neoplasias (IPMN), 12 with borderline IPMN and nine with malignant IPMN were subjected to immunohistochemistry. Fixation, sectioning and immunohistochemistry were carried out as described earlier (Hennig *et al*, 2002). The primary polyclonal antibody to BLT2 from Cayman Chemicals (Ann Arbor, MI, USA) was used at 1 : 50. The stained tissue samples were examined by two pathologists. Controls included incubation in the absence of primary antibody and quenching of the primary antibody with the respective blocking peptide for 1 h at room temperature before application to the tissue.

### Cell lines and cell culture

The cell lines used, AsPC-1, Colo357, and Panc-1 were established from patients with pancreatic adenocarcinoma. The entire human pancreatic cancer cell lines were purchased from the American Type Culture Collection (ATCC, Rockville, MA, USA) and grown in RPMI-1640 medium (Invitrogen Life Technologies, Karlsruhe, Germany), supplemented with 10% FBS (PAN-Biotech GmbH, Aidenbach, Germany) and 100 U ml^−1^ penicillin–streptomycin (Invitrogen Life Technologies, Karlsruhe, Germany).

### Real-time light cycler® quantitative polymerase chain reaction (QRT–PCR)

All reagents and equipment for mRNA/cDNA preparation were purchased from Roche Applied Science Diagnostics (Mannheim, Germany). mRNA extractions were prepared by automated isolation using the MagNA Pure LC instrument and isolation kits I (for cells) and II (for tissue). cDNA was prepared using the first strand cDNA synthesis kit (AMV) according to the manufacturer's instructions. Real-time PCR was performed with the Light Cycler Fast Start DNA SYBR Green kit. All primers were obtained from Search-LC (Heidelberg, Germany). Primers for BLT1 were: 5′-ACTGCCTCCAGCCCTCTCAA-3′ (forward) and 5′-TAGCATTCTGCCAGGAGGAAA-3′ (reverse). Primers for BLT2 were: 5′-ACCTGTAGGCCCAGAAGGATGT-3′ (forward) and 5′-GAAGTCTTCCAGCTCAGCAGTGT-3′ (reverse). The calculated number of specific transcripts was normalised to the housekeeping genes cyclophilin B and hypoxanthine guanine phosphoribosyltransferase, and expressed as number of copies per microlitre of input cDNA.

### Overexpression of BLT2 in pancreatic cancer cell lines

Human BLT2 and formyl-methionyl-leucyl-phenylalanine receptor (fMLPR) were subcloned into the pcDNA3 expression vector (Invitrogen Life Technologies, Carlsbad, CA, USA) using *Bam*HI/*Eco*RI sites. Colo357, Panc-1 and AsPC-1 cells were cultured in RPMI-1640 medium supplemented with 10% FBS. For stable expression of BLT2 or fMLPR, cells were transfected with pcDNA3-hBLT2 or pcDNA3-hfMLPR using Lipofectamine 2000^TM^ (Invitrogen) and selected with G418 (geneticin) 1.5 mg ml^−1^ (Colo357 and Panc-1) or 3 mg ml^−1^ (AsPC-1) (Promega Biosciences Inc., Mannheim, Germany). The pcDNA3 vector alone and pcDNA3-hfMLPR were used as controls. After 2 weeks, the concentration of G418 was decreased to 0.5 mg ml^−1^. Cells were lysed, RNA was isolated and the expression of pcDNA3-hBLT2 or pcDNA3-hfMLPR was confirmed by real-time RT–PCR.

### siRNA transfection

Three different sets of siRNA were tested and the most effective set knocking down BLT2 was used for our experiments. AsPC-1 and Panc-1 BLT2-transfected pancreatic cancer cells were seeded into 6-well microplates at a concentration of 1–2 × 10^4^ in RPMI-1640 supplemented with 10% FBS (complete medium). After 24 h, BLT2 siRNA (sense 5′ CCACGCAGUCAACCUUCUGtt 3′, antisense 5′CAGAAGGUUGACUGCGUGGta 3′) or negative control siRNA (AATTCTCCGAACGTGTCACGT) were added using RNAiFectTH Transfection Kit (Qiagen, Hilden, Germany) to give a final concentration of 5 *μ*g siRNA per well according to manufacturer's instructions.

Medium was changed 24 h after transfection, and cells were grown in complete culture medium.

Next, mRNA was extracted after 48 h of transfection using the MagNA Pure LC instrument and isolation kits I (for cells) for QRT–PCR analysis.

### Proliferation studies: in tumour cells overexpressing BLT2

AsPC-1, Panc-1 and Colo357 Mock, fMLPR and BLT2-transfected pancreatic cancer cells were seeded into 6-well microplates at a concentration of 1 × 10^4^ cells per well for AsPC-1, 2 × 10^4^ cells per well for Colo357 and 4 × 10^4^ cells per well for Panc-1 and incubated at 37°C in RPMI-1640 supplemented with 10% FBS. After 24, 48, 72, 96 and 120 h cells were harvested and viable cells counted using Guava PC (Guava-PC Technologies, Hayward, CA, USA). In addition, proliferation was analysed by 3-[4,5-dimethylthiazol-2-yl]-2,5-diphenyltetrazolium bromide (MTT) assay.

### Proliferation studies: in tumour cells treated with BLT2 ligands

In further studies cells were seeded into 12-well microplates in complete medium (30–50% confluence). After 24 h, cells were cultured in serum-free media with or without different concentrations of LTB_4_, Compound A and Compound B (control) for 24, 48, 72, 96, and 120 h. Medium was changed after 48 h. At the end of each time point, the cells were trypsinised to produce single cell suspension and the viable cell number in each well counted using Guava PC (Guava Technologies, Hayward, CA, USA). For MTT assay cells were seeded into 12-well microplates in complete medium at a concentration of 2 × 10^4^ cells per well. After 24 h, cells were cultured in serum-free media with or without different concentrations of LTB_4_, Compound A and Compound B (control) for 24, 48, 72 and 96 h. Medium was changed after 48 h. At the end of each time period 5 mg ml^−1^ MTT was added.

### Proliferation studies: in tumour cells treated with BLT2 siRNA

For the siRNA proliferation studies, cells were trypsinised and the viable cell number was counted at 24, 48, 72, 96 and 120 h after cell seeding using Guava PC (Guava Technologies, Hayward, CA, USA).

### Proliferation studies: in tumour cells treated with BLT2 ligands and siRNA

In an additional experiment, cells were seeded into 12-well microplates in complete medium (30–50% confluence). After 24 h, cells were cultured in serum-free medium with or without Compound A and Compound B (control) for 48 h. At this time point, the medium was changed and BLT2 siRNA (sense 5′ CCACGCAGUCAACCUUCUGtt 3′, antisense 5′CAGAAGGUUGACUGCGUGGta 3′) or negative control siRNA (AATTCTCCGAACGTGTCACGT) were added using RNAiFectTH Transfection Kit (Qiagen, Hilden, Germany) to give a final concentration of 5 *μ*g siRNA per well according to manufacturer's instructions. The medium was changed 12 h after transfection, and the cells were left to grow in complete culture medium for another 12 h. Twenty-four hours after siRNA transfection, the medium was changed and cells were grown in complete culture medium with or without Compound A and Compound B (control) for another 24 and 48 h under siRNA effect and then counted using Guava PC (Guava Technologies, Hayward, CA, USA).

## Statistical analysis

Proliferation studies have been repeated at least three times independently. Data on BLT2 expression by immunohistochemistry in humans were analysed by Kruskal–Wallis one-way ANOVA with the Dunn's method as *post hoc* test. Results from QRT–PCR were analysed using one-way ANOVA with the Student–Newman–Keul's Method as *post hoc* test for multiple paired comparisons. Paired *t*-test and Friedman repeated measures analysis of variance on ranks (with Tukey's as *post hoc* test for pairwise multiple comparisons) were used to analyse cell proliferation (cell counting).

## Results

### Expression of BLT2 in human pancreatic tissues

**Figure 1:**
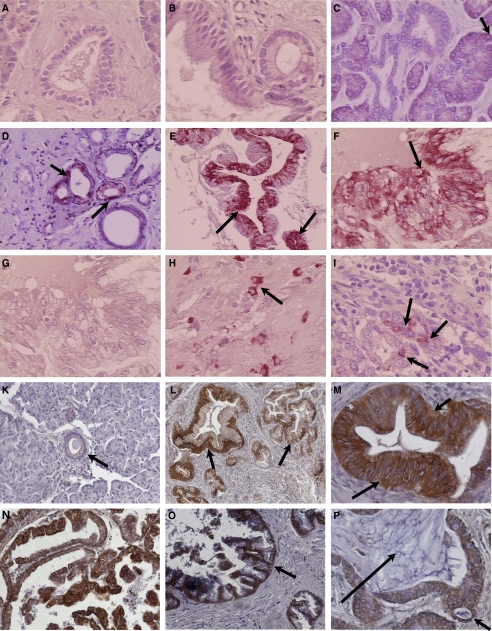
Immunohistochemical localisation of BLT2 in human pancreatic tissues. **A–I** shows normal pancreatic tissues obtained from multi-organ donors, chronic pancreatitis and pancreatic adenocarcinoma stained by the AP-Red system. **K**–**P** demonstrates staining in IPMN tissues using the DAB system. (**A**) unstained duct in normal pancreas (× 400); (**B**) unstained PanIN-1a lesion in normal pancreas (× 400); (**C**) unstained ducts in chronic pancreatitis and basal staining in some acinar cells (× 400). (**D**) PanIN-2 lesion in chronic pancreatitis with positive staining in the cytoplasm (× 200); (**E**) and (**F**) PanIN-3 lesion in pancreatic cancer with intense cytoplasmic staining (× 200, × 400); (**G**) unstained PanIN-3 lesion in an adjacent section to (**F**) but in the presence of the blocking peptide to quench the staining (× 400); (**H**) infiltrating tumour cells in pancreatic cancer with marked positive staining in the cytoplasm (× 400); (**I**) positive-stained tumour cells in a lymph node metastasis (× 400); (**K**) unstained normal duct next to an IPMN adenoma (× 200); intense cytoplasmic staining is shown in (**L**) IPMN adenoma branch duct type (× 100), (**M**) main duct type (× 400), (**N**) borderline IPMN of the oncocytic type (× 200), and in malignant invasive IPMNs of the (**O**) tubular (× 200) and (**P**) colloid type (× 400). The primary antibody to BLT2 was used at 1 : 50 on deparaffinised tissues.

BLT2 were found to be markedly upregulated in PanIN lesions and cancer cells, but were not expressed in islet cells, except in four specimens obtained from patients with chronic pancreatitis (Figure 1). PanIN-1a lesions and normal ductal cells showed absolutely no staining for BLT2, however, we observed strong positive staining in all PanIN-1b, two and three lesions which were found in 8 of 10 pancreatic adenocarcinoma tissues and in two specimens from patients with chronic pancreatitis (Figure 1). The other eight chronic pancreatitis tissues contained either normal ducts and/or PanIN-1a lesions, which did not stain for BLT2. Furthermore, infiltrating tumour cells showed strong positive staining in pancreatic adenocarcinoma tissues and in a lymph node metastasis (Figure 1). Acinar cells in normal pancreas, chronic pancreatitis and pancreatic adenocarcinoma sporadically showed BLT2 expression in the cytoplasm adjacent to the basolateral membrane. Details are shown in Table 1.

In addition, immunohistochemistry was performed in a variety of pancreatic tissues showing IPMN lesions. Moderate (12 of 28) or strong (13 of 28) cytoplasmic staining for BLT2 was detected in 25 of 28 tissues with IPMNs regardless of whether they were benign or malignant or if they are main or branch duct type lesions (Figure 1). Acinar cells did not stain for BLT2. Islet cells, when applicable, showed weak (16 of 28) or moderate (3 of 28) expression of BLT2 in their cytoplasm. Details are shown in Table 2. Negative controls for BLT1 and BLT2 (first antibodies quenched with the appropriate blocking peptides) showed no staining (Figure 1). In most sections, inflammatory cells stained positive for BLT2 and this provided a valuable internal control. In normal tissues and tissues containing only PanIN1a lesions, these were the only cells with significant staining. It is likely that these were mast cells, but we did not confirm this by double staining.

**Figure 2:**
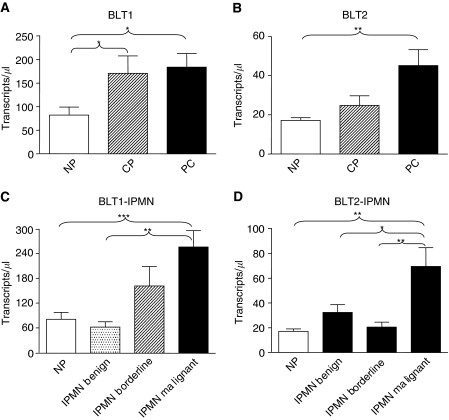
Expression of BLT1 and BLT2 on mRNA level in human pancreatic tissues using real-time RT–PCR (NP=normal pancreas obtained from multi-organ donors, CP=chronic pancreatitis, PC=pancreatic adenocarcinoma, IPMN=intraductal papillary mucinous neoplasia). (**A**) shows BLT1 expression in CP and PC; (**B**) shows BLT2 in CP and PC; (**C**) shows BLT1 in IPMNs and (**D**) shows BLT2 in IPMNs. ^*^P<0.05; ^**^P<0.01; ^***^P<0.001.

The results obtained from QRT–PCR confirmed the immunohistochemical findings. BLT1 and BLT2 mRNA were upregulated in chronic pancreatitis and malignant pancreatic tissues. BLT1 expression is approximately fivefold higher than BLT2. Furthermore, although BLT2 is significantly overexpressed only in malignant pancreatic tissues, BLT1 is also significantly upregulated in tissues from patients with chronic pancreatitis (Figure 2).

### Expression of BLT1 and BLT2 in human pancreatic cancer cells

**Figure 3:**
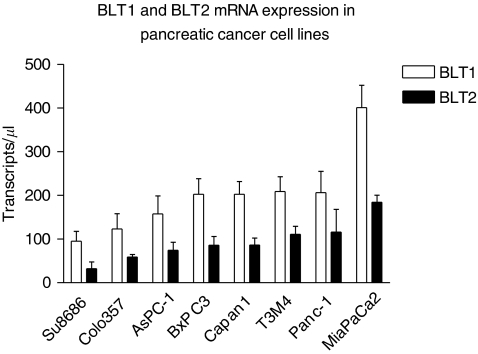
Expression of BLT1 and BLT2 on mRNA level in pancreatic cancer cell lines using real-time RT–PCR.

Both, BLT1 and BLT2 were found to be expressed at the mRNA level in all human pancreatic cancer cell lines (MiaPaCa2, AsPC-1, T3M4, Panc-1, Su8686, Capan1, BxPC3, Colo357) tested. Real-time RT–PCR revealed that expression levels of both receptors differed between cell lines, with approximately 2-fold higher BLT1 mRNA expression compared with BLT2 expression (*P*<0.001). MiaPaCa2 cells showed the highest expression for both receptors compared with the other cell lines (BLT1 all *P*<0.005; BLT2 all *P*<0.05) (Figure 3).

### Overexpression and knockdown of BLT2 and its role on tumour cell proliferation

**Figure 4:**
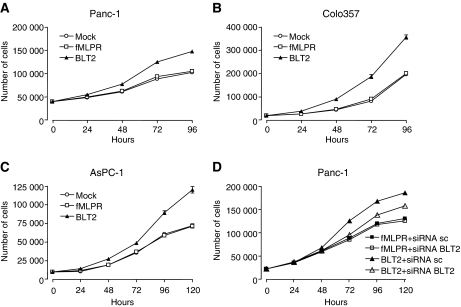
The effect of overexpression of BLT2 in Panc-1 (**A**), Colo357 (**B**) and AsPC-1 (**C**) cells on proliferation of these cells, evaluated by cell counting over 96 and 120 h. Significant growth stimulation is shown for BLT2-transfected cells compared with Mock and fMLPR (*P*<0.001). Reversibility of this effect after siRNA transfection is demonstrated for Panc-1 cells by cell counting over 120 h (**D**). (*P*<0.001 for BLT2 siRNA sc *vs* BLT2 siRNA BLT2 and BLT2 siRNA sc *vs* fMLPR).

The effect of stable overexpression of BLT2 on cell proliferation was studied in AsPC-1, Panc-1 and Colo357 cells (AsPC-1-BLT2, Panc-1-BLT2 and Colo357-BLT2 cells, respectively). Receptor mRNA levels increased after BLT2 transfection from 50 to 4176 transcripts per microlitre in Colo357, from 95 to 11 310 transcripts per microlitre in Panc-1 and from 142 to 1004 transcripts per microlitre in AsPC-1 cells. BLT2 mRNA levels remained stable on the basal expression level after transfection with hfMLPR or the empty pcDNA3 vector (mock). Proliferation over 96 and 120 h was significantly increased in Panc-1-BLT2, Colo357-BLT2 and AsPC-1-BLT2 cells, respectively compared with Panc-1-hfMLPR, Colo357-hfMLPR and AsPC-1-hfMLPR cells as well as their mock transfected clones, measured by cell counting (*P*<0.001) (Figure 4) and MTT assay (*P*<0.001) (data not shown).

**Figure 5:**
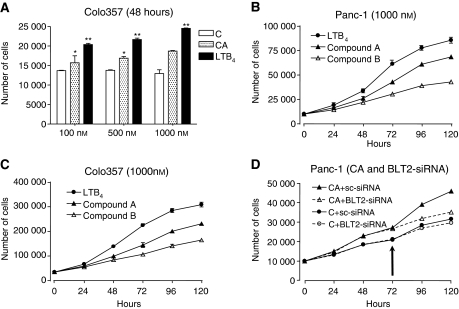
Treatment with LTB_4_ (BLT1 and BLT2 agonist) or Compound A (selective BLT2 agonist) significantly increased proliferation of Panc-1 and Colo357 cells dose (over 48 h; ^*^P<0.05; ^**^P<0.01) and time dependently (at 1000 nM; *P*<0.001 for LTB_4_ and Compound A *vs* Compound B). Compound B is a methyl ester and served as negative control. Dose-dependent effects are shown in (**A**) and time-dependent effects in (**B**) and (**C**). Panel (**D**) demonstrates significant growth inhibition of Panc-1 cells (not overexpressing BLT2) by BLT2 siRNA when BLT2 is stimulated with Compound A. The black arrow marks when siRNA was added.

The effect of BLT2 overexpression on tumour cell proliferation was inhibited by siRNA transfection. After 48 h BLT2 mRNA levels decreased from 2583 to 802 transcripts per microlitre in Panc-1 and from 2198 to 830 transcripts per microlitre in AsPC-1 cells, causing significant growth inhibition of Panc-1-BLT2 and AsPC-1-BLT2 cells (*P*<0.001). Despite several attempts, we were not able to successfully transfect Colo357 cells with BLT2 siRNA. Transfection of scrambled siRNA as control did not change the proliferation of Panc-1-BLT2, AsPC-1-BLT2, Panc-1-hfMLPR and AsPC-1-hfMLPR cells. Data are illustrated for Panc-1 in Figure 4. In tumour cells that do not overexpress BLT2, we observed significant growth inhibition by BLT2 siRNA when BLT2 was stimulated with Compound A but not without stimulation (Figure 5).

### Effects of BLT2 agonists on tumour cell proliferation

The selective synthetic BLT2 agonist, Compound A, caused significant growth stimulation of pancreatic cancer cells in a concentration- and time-dependent manner. The final dose of 1000 nM we used in our time-dependent experiments might be high, but is reasonable as the metabolism and half-life of Compound A are not known. Compound B, its methyl ester derivative without any stimulatory effects on BLT2 (Iizuka *et al*, 2005) used as a negative control, did not alter proliferation of these tumour cells. Treatment of pancreatic cancer cells with the common BLT1 and BLT2 agonist LTB_4_ caused an additional growth advantage when compared with cells treated with Compound A or B. Proliferation was measured by cell counting and MTT assay. Data for the treatment of Colo357 and Panc-1 cells are shown in Figure 5.

## Discussion

Arachidonic acid is the precursor of eicosanoids, which are important mediators involved in inflammation as well as in the growth of different cancers, including colon, prostate, breast, lung and pancreatic adenocarcinoma (Ding *et al*, 2003). There are two major groups of eicosanoids: (1) prostaglandins, that are produced by cyclooxygenases and (2) leukotrienes, that are generated by 5-lipoxygenase (5-LOX). Leukotrienes are potent pro-inflammatory mediators that have important functions in inflammatory disorders and allergies (Sampson, 2000; Shao *et al*, 2006). Recently, we and others have demonstrated that leukotrienes may also be important in cancer development, metastases and cachexia (Paruchuri *et al*, 2002, 2006; Tong *et al*, 2002). In the BOP-hamster model leukotriene concentration was significantly increased in pancreatic carcinoma when compared with tumour-free tissue (Heukamp *et al*, 2006). Moreover, fish oil (rich in n-3 PUFAs) decreased the incidence of liver metastases, possibly because of decreased intrametastatic leukotriene concentration (Heukamp *et al*, 2006). Expression of the leukotriene D_4_ receptor (CysLT_1_) is increased in colorectal adenocarcinomas and leukotriene D_4_ (LTD_4_) and LTB_4_ stimulate colon cancer cell proliferation by activating ERK1/2 (Paruchuri *et al*, 2002, 2006; Nielsen *et al*, 2003, 2005; Ohd *et al*, 2003). Furthermore, inhibitors of leukotriene production can effectively prevent the lung cancer development in mice (Gunning *et al*, 2002). We have recently shown, that LTB_4_ stimulates pancreatic cancer cell growth by activating ERK1/2; an effect inhibited by the unspecific LTB_4_ receptor antagonist, LY293111 (Tong *et al*, 2002, 2007). We previously demonstrated the expression of BLT1 in human pancreatic cancer tissues with strong staining in cancer cells and the islets surrounding these tumours (Hennig *et al*, 2002). BLT2 mRNA expression was demonstrated in murine and human mast cells responsible for recruitment and accumulation of mast cells in areas of inflammation (Lundeen *et al*, 2006). Another study described a TNFα-dependent expression of BLT2 in human umbilical vein endothelial cells which may be important during early vascular responses to inflammation (Qiu *et al*, 2006). In addition, greatly increased BLT2 mRNA levels were found in a variety of human cancers by *in situ* hybridisation (Yoo *et al*, 2004). Pancreatic specimens were not a subject of this study (Yoo *et al*, 2004). Yoo *et al* (2004) suggested that a LTB_4_-BLT2-linked cascade has a crucial mediatory function in cell transformation induced by oncogenic Ha-Ras^V12^. LY255283 has been described as a selective BLT2 antagonist (Qiu *et al*, 2006; Shin *et al*, 2006). However, other results demonstrate unspecificity of LY255283 for BLT2. Until now, no specific BLT2 inhibitor is available. Very recently, Choi *et al* (2007) could demonstrate that BLT2 is a key regulator of ROS in H-Ras^V12^-transformed fibroblasts and suggested a LTB_4_-BLT2-Nox1-linked cascade responsible for elevated ROS generation in Ras-transformed cells. However, there is still limited information on the physiological role of BLT2 in cancer and this receptor has not been previously linked to pancreatic cancer. The current results demonstrate the expression of BLT2 in all human pancreatic cancer cell lines tested and overexpression in all the human pancreatic tissues obtained from patients with pancreatic adenocarcinoma and chronic pancreatitis, when PanIN lesions are present. BLT2 is also upregulated in IPMNs. Strong BLT2 staining was seen in all grades of PanINs, except PanIN-1a. BLT2 staining was also intense in benign and malignant IPMNs; another progression model for pancreatic cancer. BLT2 was significantly upregulated in malignant IPMNs when compared with benign IPMNs and normal pancreatic specimens. However, all IPMNs, including the benign lesions showed specific staining for BLT2. This might be explained by the fact that complete tumour specimens and not microdissected IPMNs were subjected to quantitative RT–PCR and benign IPMNs are often smaller than malignant IPMNs.

Overexpression of BLT2 in three different human pancreatic cancer cell lines resulted in increased proliferation compared with control cells transfected with either hfMLPR or empty vector. This is consistent with our previous findings that LTB_4_ stimulates pancreatic cancer cell growth (Tong *et al*, 2002). Pancreatic cancer cells secrete LTB_4_ and produce growth factors such as epidermal growth factor (EGF). In addition, EGF is able to stimulate LTB_4_ production and secretion in these cells. Therefore, LTB_4_ may be involved in the autocrine growth stimulation of pancreatic cancer cells acting on both receptors. This might explain why recombinant overexpression of BLT2 in cancer cells already expressing endogenous BLT2 have a growth advantage without any further treatment, because more receptor-binding sites (BLT2) can be stimulated by LTB_4_. Moreover, serum contains so far unspecified lipid factors stimulating BLT2. Furthermore, LTB_4_ has previously been linked to insulin secretion. As we observed that BLT1 is upregulated in islets adjacent to pancreatic adenocarcinoma, it is tempting to speculate that the tumours may induce additional paracrine growth stimulation by augmenting local insulin secretion in the pancreas (Pek and Walsh, 1984; Hennig *et al*, 2002).

Selective BLT2 stimulation with Compound A and inhibition with siRNA caused increased tumour cell proliferation or growth inhibition, respectively. However, siRNA treatment of cancer cells expressing endogenous BLT2 did not affect their proliferation rate. This is difficult to fully explain, because if endogenous LTB_4_ stimulates growth through BLT2 receptors, then receptor knockdown would be expected to reduce proliferation. However, the answer may lie in the fact that both receptor subtypes respond to this ligand. Reduction in copy number of BLT2 may merely allow the LTB_4_ to signal through the BLT1, which have higher affinity for the ligand. However, in cells with upregulated BLT2, knockdown will reveal the growth effects of this receptor. Evidence that this explanation is correct comes from the experiments with Compound A in cells (a compound that is relatively specific for BLT2) without overexpression of BLT2. Here, the BLT2 knockdown did block proliferation.

As BLT2 are not or only weakly expressed in normal ductal cells, their marked upregulation in PanINs, IPMNs and infiltrating cancer cells suggests a beneficial role of this receptor for the cancer cells. To our knowledge, this is the first report linking BLT2 to pancreatic cancer. We may have found a selective PanIN and IPMN marker. Maitra *et al* (2002) reported that COX-2 is overexpressed in 65% of PanIN lesions and suggested the use of COX-inhibitors for chemoprevention in patients at high risk. However, COX-2 is not expressed in all PanIN lesions and is also expressed in islets (Maitra *et al*, 2002). BLT2 may prove to be a superior target, because of the consistent and selective expression in PanINs and IPMNs. We speculate that upregulation of BLT2 is associated with ductal changes. However, we do not claim BLT2 as a specific marker for pancreatic carcinogenesis. BLT2 could be considered as an imaging target for early neoplastic lesions, for example, in patients at high risk for pancreatic cancer. The expected background signal in normal pancreas should be sufficiently low, because acinar and islet cells only showed weak positive staining in some tissues and BLT2 mRNA levels in whole pancreas are very low compared with other organs. Finally, development of a combined BLT1 and BLT2 antagonist (Hicks *et al*, 2007) could be a valuable approach for the treatment and chemoprevention of pancreatic and perhaps other cancers.

## Figures and Tables

**Table 1:** Immunohistochemistry for BLT2 in pancreatitis and pancreatic cancer

**Sample**	**Normal ducts**	**PanIN**	**Cancer cells**	**Islet cells**	**Acini**
*Normal (c.d.)*					
1	Negative	Absent	Absent	Negative	+
2	Negative	Absent	Absent	Negative	+
3	Negative	1a: negative	Absent	Negative	+
4	Negative	Absent	Absent	Negative	+
5	Negative	Absent	Absent	Negative	+
6	Negative	Absent	Absent	+	+
7	Negative	Absent	Absent	Negative	Negative
8	Negative	Absent	Absent	Negative	+
9	Negative	Absent	Absent	+	+
10	Negative	Absent	Absent	Negative	Negative
					
*CP*					
1	Negative	1b: +++	Absent	+++	+
2	Negative	Absent	Absent	+	Negative
3	Negative	Absent	Absent	Negative	Absent
4	Negative	Absent	Absent	+	Negative
5	Negative	Absent	Absent	Negative	Negative
6	Negative	Absent	Absent	Negative	Negative
7	Negative	Absent	Absent	Negative	Negative
8	Negative	1a: negative	Absent	+	+
9	Negative	1a: negative; 1b: ++	Absent	Negative	Negative
10	Negative	1a: negative	Absent	+	Negative
					
*PC*					
1	Absent	3: +++	++	Negative	+
2	Negative	3: +++	++	Negative	+
3	Negative	1b, 2, 3: +++	++	Absent	Absent
4	Negative	Absent	+++	Negative	Negative
5	Absent	1a: negative, 1b: ++	Absent	Absent	Absent
6	Negative	Absent	+++	Negative	Negative
7	Negative	2: +++	Absent	Negative	+
8	Negative	2, 3: +++	+++	Negative	+
9	Negative	2, 3: +++	+++	Negative	Negative
10	Negative	2, 3: +++	+++	Negative	Negative
					
Kruskal–Wallis ANOVA	No difference	*P*<0.001	*P*<0.001	No difference	No difference
Normal *vs* CP		*P*=0.252	*P*=1		
Normal *vs* PC		*P*<0.001	*P*<0.001		
CP *vs* PC		*P*<0.001	*P*<0.001		

c.d.=cadaver donor; CP=chronic pancreatitis; PC=pancreatic cancer.On account of the multiple groups, statistical analysis in the human samples was carried out by Kruskal–Wallis one-way ANOVA with Dunn's method as *post hoc* test for multiple comparisons. PanIN: pancreatic intraepithelial neoplasia, grade 1a, 1b, 2 and 3.

Absent: described structures are not present in this tissue section.

Negative: unstained but present structures.

‘+’ Weak positive staining.

‘++’ Positive staining.

‘+++’ Strong positive staining.

**Table 2:** Immunohistochemistry for BLT2 in IPMNs

**Sample**	**Normal ducts**	**IPMN**	**Cancer cells**	**Islet cells**	**Acini**
*IPMN-adenoma*					
1	+	+	Absent	Negative	Negative
2	+	+++	Absent	+	Negative
3	+	+++	Absent	+	Negative
4	Negative	++	Absent	+	Negative
5	+	+++	Absent	+	Negative
6	+	+++	Absent	++	Negative
7	++	++	Absent	++	Negative
					
*IPMN-borderline*					
1	+	++	Absent	+	Negative
2	+	++	Absent	+	Negative
3	+	++	Absent	+	Negative
4	+	+++	Absent	+	Negative
5	++	++	Absent	+	Negative
6	+	+	Absent	+	Negative
7	++	+++	Absent	++	Negative
8	+	++	Absent	+	Negative
9	+	+++	Absent	Absent	Absent
10	+	+++	Absent	+	Absent
11	+	++	Absent	+	Negative
12	++	+++	Absent	+	Negative
					
*IPMN-malignant*					
1	Absent	++	++	Absent	Absent
2	Absent	+++	+++	Absent	Absent
3	Absent	+++	+++	Absent	Absent
4	+	+++	+++	+	Absent
5	+	+++	+++	+	Negative
6	Absent	++	++	Absent	Absent
7	Absent	++	++	Absent	Absent
8	Absent	++	++	Absent	Absent
9	Absent	Negative	Negative	Absent	Absent
					
Kruskal–Wallis ANOVA	No difference	*P*=0.896	*P*<0.001	No difference	No difference
Adenoma *vs* borderline			*P*=1		
Adenoma *vs* malignant			*P*<0.001		
Borderline *vs* malignant			*P*<0.001		

IPMN=intraductal papillary mucinous neoplasia.

On account of the multiple groups, statistical analysis in the human samples was carried out by Kruskal–Wallis one-way ANOVA with Dunn's method as *post hoc* test for multiple comparisons. Absent: described structures are not present in this tissue section.

Negative: unstained but present structures.

‘+’ Weak positive staining.

‘++’ Positive staining.

‘+++’ Strong positive staining.
